# A Comparison of Regularization Methods in Forward and Backward Models for Auditory Attention Decoding

**DOI:** 10.3389/fnins.2018.00531

**Published:** 2018-08-07

**Authors:** Daniel D. E. Wong, Søren A. Fuglsang, Jens Hjortkjær, Enea Ceolini, Malcolm Slaney, Alain de Cheveigné

**Affiliations:** ^1^Laboratoire des Systèmes Perceptifs, CNRS, UMR 8248, Paris, France; ^2^Département d'Études Cognitives, École Normale Supérieure, PSL Research University, Paris, France; ^3^Department of Electrical Engineering, Danmarks Tekniske Universitet, Kongens Lyngby, Denmark; ^4^Danish Research Centre for Magnetic Resonance, Copenhagen University Hospital Hvidovre, Hvidovre, Denmark; ^5^Institute of Neuroinformatics, University of Zürich, Zurich, Switzerland; ^6^AI Machine Perception, Google, Mountain View, CA, United States; ^7^Ear Institute, University College London, London, United Kingdom

**Keywords:** temporal response function, speech decoding, electroencephalography, selective auditory attention, attention decoding

## Abstract

The decoding of selective auditory attention from noninvasive electroencephalogram (EEG) data is of interest in brain computer interface and auditory perception research. The current state-of-the-art approaches for decoding the attentional selection of listeners are based on linear mappings between features of sound streams and EEG responses (forward model), or vice versa (backward model). It has been shown that when the envelope of attended speech and EEG responses are used to derive such mapping functions, the model estimates can be used to discriminate between attended and unattended talkers. However, the predictive/reconstructive performance of the models is dependent on how the model parameters are estimated. There exist a number of model estimation methods that have been published, along with a variety of datasets. It is currently unclear if any of these methods perform better than others, as they have not yet been compared side by side on a single standardized dataset in a controlled fashion. Here, we present a comparative study of the ability of different estimation methods to classify attended speakers from multi-channel EEG data. The performance of the model estimation methods is evaluated using different performance metrics on a set of labeled EEG data from 18 subjects listening to mixtures of two speech streams. We find that when forward models predict the EEG from the attended audio, regularized models do not improve regression or classification accuracies. When backward models decode the attended speech from the EEG, regularization provides higher regression and classification accuracies.

## 1. Introduction

A fundamental goal of auditory neuroscience is to understand the mapping between auditory stimuli and the cortical responses they elicit. In magneto/electro-encephalography (M/EEG) studies, this mapping has predominantly been measured by examining the average cortical evoked response potential (ERP) to a succession of repeated short stimuli. More recently, these methods have been extended to continuous stimuli such as speech by using linear system-response models, broadly termed “temporal response functions” (TRFs), that are estimated using system-identification methods. The TRF is a stimulus-response model that characterizes how a unit impulse in an input feature corresponds to a change in the M/EEG data. TRFs can be used to generate continuous predictions about M/EEG responses as opposed to characterizing the response (ERP) to repetitions of the same stimuli. Importantly, it has been demonstrated that the stimulus-response models can be extracted both from EEG responses to artificial sound stimuli (Lalor et al., 2006, 2009; Power et al., 2011) but also from EEG responses to naturalistic speech (Lalor and Foxe, 2010). A number of studies have considered mappings between the slowly varying temporal envelope of a speech sound signal (<10 Hz) and the corresponding filtered M/EEG response (Lalor and Foxe, 2010; Ding and Simon, 2012a,b, 2013, 2014). However, TRFs are not just limited to the broadband envelope, but can also be obtained with the speech spectrogram (Ding and Simon, 2012a,b), phonemes (Di Liberto et al., 2015), or semantic features (Broderick et al., 2018). This has opened new avenues of research into cortical responses to speech, advancing the field beyond examining responses to repeated isolated segments of speech.

TRF methods have proven particularly apt for studying how the cortical processing of speech features are modulated by selective auditory attention. A number of studies have considered multi-talker “cocktail party” scenarios, where a listener attends to one speech source and ignores others. It has been demonstrated that both attended and unattended acoustic features can be linearly mapped to the cortical response (Ding and Simon, 2012a,b; Power et al., 2012; Zion Golumbic et al., 2013; Puvvada and Simon, 2017).

Conversely, the same linear model, which maps speech features to the cortical response (forward direction), can be adapted to provide a linear mapping from the cortical response to the speech features (backward direction) (Bialek et al., 1991; Mesgarani et al., 2009; Ding and Simon, 2012a,b; Mesgarani and Chang, 2012; Mirkovic et al., 2015; O'Sullivan et al., 2015; Fuglsang et al., 2017; Van Eyndhoven et al., 2017). The mapping from acoustic features to cortical responses is typically referred to as a forward model (or TRF), whereas the mapping from cortical responses to acoustic features is referred to as a backward model (Haufe et al., 2014). The quality of model fit reflects the degree to which cortical activity is driven by stimulation. In a cocktail party scenario, the quality of fit between each of the speech streams and the cortical activity can be used to infer which speech stream is being attended. Differences in the accuracy of forward/backward model-derived estimates between the attended and unattended speech signal can be used to predict or “decode” to whom a listener is attending based on unaveraged M/EEG data. Single-trial measures of auditory selective attention in turn suggests BCI applications, for instance, for cognitively-steered hearing aids (Das et al., 2016; O'Sullivan et al., 2017; Van Eyndhoven et al., 2017; Zink et al., 2017).

The ability of forward/backward stimulus-response models to generalize to new data is generally limited by the need to estimate a relatively large number of parameters based on noisy single-trial M/EEG responses. Like many aspects of machine learning, this necessitates regularization techniques that constrain the model coefficients to prevent overfitting (Crosse et al., 2016a; Holdgraf et al., 2017). A number of methods for regularizing the forward/backward stimulus-response models have been presented in various studies (Goutte et al., 2000; Theunissen et al., 2000, 2001; Machens et al., 2004; David et al., 2007; Thorson et al., 2015). Each of these methods attempt to address the challenge of having sufficient data to compute a reliable stimulus-response mapping function. To reduce the data requirement, regularization can be applied in the form of a smoothness and/or sparsity constraint.

To date, little work has been done to compare these methods against each other. A meta-analysis would be difficult as many variables, such as subjects, stimuli and data processing are different between each study. The present paper uses a standardized publicly available dataset^1^ (Fuglsang et al., 2018), based on the attended-vs.-unattended talker discrimination task, as well as preprocessing and evaluation procedures to compare these algorithms. In addition, the present paper examines the relationship between different evaluation metrics to highlight their similarities and differences. The methods for computing forward/backward stimulus-response models have been implemented in the publicly available Telluride Decoding Toolbox^2^.

## 2. Materials and methods

Temporal response functions can be used to predict the EEG response to a multi-talker stimulus from the attended speech envelope or, alternatively, the equation can be adapted to reconstruct the attended speech envelope from the EEG response. The first case is denoted as a “forward model” (as it maps from speech features to neural data) and the second as a “backward model” (as it maps from neural data back to speech features) (Haufe et al., 2014).

### 2.1. Stimulus-response models

The linear stimulus-response models below described below map a matrix **X** (stimulus features for a forward model, EEG for a backward model) to a matrix **Y** (EEG channels for a forward model, stimulus features for a backward model):

(1) $$\begin{array}{l} \hat{\text{Y}}=\text{X}\text{W}, \end{array}$$

where **X** = [*x*_*t*, (*f, c*)_] is a multichannel data matrix (channels indexed by *c*), augmented to include time-lagged versions of the data (lags indexed by *f*), and Ŷ = [*y*_*t*_] is the model estimate in the form of a vector indexed by time *t*. Time lags, limited to a range such as -500 to + 500 ms, allow the model to handle delays and convolutional mismatch between **X** and **Y**. Dimensions *c* and *f* are combined when performing matrix multiplications.

In the following subsections we introduce different approaches to estimating the linear model parameters, **W**. Each method uses different regularization techniques to optimize the generalizability of the mapping functions.

#### 2.1.1. Ordinary least squares (OLS)

The cost function that is minimized when solving the regression model is:

(2) $$\begin{array}{l} L\left(\text{W}\right)={\left(\text{Y}-\text{X}\text{W}\right)}^{T}\left(\text{Y}-\text{X}\text{W}\right). \end{array}$$

The filter coefficients of this model can be estimated via ordinary least squares:

(3) $$\begin{array}{l} \text{W}={\left({\text{X}}^{T}\text{X}\right)}^{-1}{\text{X}}^{T}\text{Y}, \end{array}$$

where **X**^*T*^**X** is the estimated autocovariance matrix and **X**^*T*^**Y** is the estimated cross-covariance matrix. The ordinary least-squares solution was here estimated using the Cholesky decomposition method, via the *mldivide* routine in Matlab. One advantage of the OLS estimator is that it has no additional hyperparameters that must be optimized. However, in practice the OLS estimator is often outperformed by the regularized solutions described in the following subsections. This is often the case when the regressor, **X**, is high-dimensional and has a poorly estimated covariance matrix given limited amounts of training data, or contains auto-correlations and/or cross-channel correlations resulting in a low rank matrix. In other words, the inverse problem is ill-posed. Such is the case when using non-stochastic data for **X**, such as speech or EEG data.

If **X** were white and standardized, the autocovariance matrix would be a multiple of the identity matrix, and the OLS and regularized approaches reduce to a straight-forward cross-correlation, also known as reverse correlation (Ringach and Shapley, 2004).

#### 2.1.2. Ridge

Ridge regression minimizes the residual sum of squares, but adds an *L*2 constraint on the regression coefficients (Machens et al., 2003; Crosse et al., 2015; Di Liberto et al., 2015; Crosse et al., 2016b; Holdgraf et al., 2016; O'Sullivan et al., 2017; Broderick et al., 2018). An *L*2 constraint smooths the regression weights by penalizing the square of the weights in **W** with a regularization constant λ for the Ridge regression cost function:

(4) $$\begin{array}{l} L{\left(\text{W}\right)}_{\lambda }={\left(\text{Y}-\text{X}\text{W}\right)}^{T}\left(\text{Y}-\text{X}\text{W}\right)+\lambda {\text{W}}^{T}\text{W} \end{array}$$

(Hastie et al., 2001; Machens et al., 2004). Ridge regression corresponds to imposing a Gaussian prior on the filter coefficients (Wu et al., 2006). The Ridge solution is:

(5) $$\begin{array}{l} \text{W}={\left({\text{X}}^{T}\text{X}+\lambda \text{I}\right)}^{-1}{\text{X}}^{T}\text{Y}, \end{array}$$

where λ is the regularization parameter that controls the amount of parameter shrinking.

#### 2.1.3. Low-rank approximation (LRA)

The LRA-based regression relies on a low-rank approximation of the covariance matrix, **X**^*T*^**X**. This is achieved by employing a singular value decomposition (SVD) of **X**^*T*^**X**:

(6) $$\begin{array}{l} {\text{X}}^{T}\text{X}=\text{U}\text{S}{\text{V}}^{T}, \end{array}$$

where **U** and **V** are orthonormal matrices that contain respectively the left and right singular vectors, and where **S** is a diagonal matrix, **S** = diag(*s*_1_, *s*_2_, ..*s*_*d*_) with sorted diagonal entries. Since **X**^*T*^**X** is a positive semidefinite matrix we have **U** = **V**. LRA uses a rank-*K* approximation of **X**^*T*^**X** by only retaining the first 1 ≤ *K* ≤ *d* diagonal elements of **S**. The cost function is:

(7) $$\begin{array}{l} L{\left(\text{W}\right)}_{K}={\left(\text{Y}-\text{X}\text{W}\right)}^{T}\left(\text{Y}-\text{X}\text{W}\right)\\ -{\text{W}}^{T}{\text{V}}_{K+1\ldots d}{\text{S}}_{K+1\ldots d,K+1\ldots d}{\text{V}}_{K+1\ldots d}^{T}\text{W}, \end{array}$$

where **V**_*K*+1…*d*_ are the *K* + 1…*d* columns of **V** and **S**_*K*+1…*d, K*+1…*d*_ is the square matrix formed by taking the *K* + 1…*d* rows and columns of **S**. By forming ${\hat{\text{S}}}^{-1}=\text{diag}\left(1/s_{1},1/s_{2},\ldots ,1/s_{K},0..0,0,0\right)$, the regression coefficients can be estimated from:

(8) $$\begin{array}{l} \text{W}=\left(\text{U}{\hat{\text{S}}}^{-1}{\text{V}}^{T}\right){\text{X}}^{T}\text{Y}. \end{array}$$

The number of diagonal elements, *K*, to retain are typically chosen such that a diagonal element is retained if the sum of the eigenvalues to be kept cover a fraction λ of the overall sum, or $0<\frac{\overset{K}{\underset{i=1}{\sum }}s_{i}}{\overset{d}{\underset{i=1}{\sum }}s_{i}}<\lambda \leq 1$. Note that the regularization parameter, λ, here is analogous to λ for Ridge Regression, but that the values are not comparable between the two. LRA is the term used in systems identification (Marconato et al., 2014), however, this type of regression has also been referred to as normalized reverse correlation (NRC) in auditory neuroscience literature (Theunissen et al., 2000, 2001; David et al., 2004, 2007; Mesgarani et al., 2009; Mesgarani and Chang, 2012).

#### 2.1.4. Shrinkage

Shrinkage (Friedman, 1989; Blankertz et al., 2011) is a method used for biasing the covariance matrix by flattening its eigenvalue spectrum with some tuning parameter, λ. In the context of regression, the Shrinkage cost function is:

(9) $$\begin{array}{l} L{\left(\text{W}\right)}_{\lambda }={\left(\text{Y}-\text{X}\text{W}\right)}^{T}\left(\text{Y}-\text{X}\text{W}\right)+\lambda {\text{W}}^{T}\left(\nu \text{I}-{\text{X}}^{T}\text{X}\right)\text{W}, \end{array}$$

where ν is here defined as the average eigenvalue trace of the covariance matrix (**X**^*T*^**X**). The solution for the cost function is:

(10) $$\begin{array}{l} \text{W}={\left(\left(1-\lambda \right){\text{X}}^{T}\text{X}+\lambda \nu \text{I}\right)}^{-1}{\text{X}}^{T}\text{Y}. \end{array}$$

When λ = 0, it becomes the standard ordinary least squares solution. When λ = 1, the covariance estimator becomes diagonal (i.e., it becomes spherical), reducing the Shrinkage equation to a cross-correlation (Blankertz et al., 2011).

These regularization schemes are related. Whereas Ridge Regression and Shrinkage both penalize extreme eigenvalues in a smooth way, LRA discards eigenvalues. Ridge and Shrinkage in other words flatten out the eigenvalue trace. Ridge shifts it up, and Shrinkage shrinks it toward an average value ν (Blankertz et al., 2011), whereas LRA cuts if off.

#### 2.1.5. Tikhonov

The scheme that we shall refer to as *Tikhonov regularization*, is a first-derivative type of Tikhonov regularization (Tikhonov, 1963) that takes advantage of the fact that there is usually a strong correlation between adjacent columns of **X** when **X** includes time shifts, because of the strong serial correlation of the stimulus envelope (for the forward model) or the filtered EEG (for the backward model). In other words, Tikhonov regularization imposes *temporal smoothness* on the model. Tikhonov regularization achieves temporal smoothness by putting a constraint in the derivative of the filter coefficients (Goutte et al., 2000; Lalor et al., 2006; Lalor and Foxe, 2010; Crosse et al., 2015, 2016a). Here we focus on first order derivatives of the filter coefficients and assume that the first derivatives can be approximated by $\frac{\partial w_{i}}{\partial i}\approx \left(w_{i+1}-w_{i}\right)$ for any neighboring filter pairs *w*_*i*+1_ and *w*_*i*_. This type of regularization is more generally referred to as 1st order Tikhonov regularization as it attempts to constrain the first derivative of the filter via central difference approximations. This gives the cost function:

(11) $$\begin{array}{l} L{\left(\text{W}\right)}_{\lambda }={\left(\text{Y}-\text{X}\text{W}\right)}^{T}\left(\text{Y}-\text{X}\text{W}\right)+\lambda \underset{i}{\sum }{\left(w_{i}-w_{i+1}\right)}^{2}. \end{array}$$

Tikhonov regularized model filters can, under this approximation, be implemented as:

(12) $$\begin{array}{l} \text{W}={\left({\text{X}}^{T}\text{X}+\lambda \text{M}\right)}^{-1}{\text{X}}^{T}\text{Y}, \end{array}$$

where

$$\begin{array}{l} \text{M}=\left[\begin{array}{cccccc} 1 & -1 & 0 & 0 & \cdots & 0\\ -1 & 2 & -1 & 0 & \cdots & 0\\ 0 & -1 & 2 & -1 & \cdots & 0\\ \vdots & \vdots & \vdots & \vdots & \; & \vdots \\ 0 & 0 & 0 & -1 & 2 & -1\\ 0 & 0 & 0 & 0 & -1 & 1 \end{array}\right]. \end{array}$$

Note that cross-channel leakage can occur whenever the regressor, **X**, reflects data recorded from multiple channels, as is the case with the backward model. This means that filter endpoints can be affected by neighboring channels as a result of the off-diagonal elements in the **M** matrix. Due to the potential for cross-channel leakage, Tikhonov has been primarily used for the forward modeling case (Crosse et al., 2016a). Despite the potential problems associated with cross-channel leakage, we also report results obtained with Tikhonov regularization for the backward model for completeness.

#### 2.1.6. Elastic net

Whereas the aforementioned regularization techniques often show improvements over the ordinary least regression in terms of generalizability, they tend to preserve all regressors in the models. This can e.g., result in nonzero filter weights assigned to irrelevant features. Lasso regression attempts to overcome this issue by putting an L1-constraint on the regression coefficients (Tibshirani, 1996). This serves to drive unnecessary coefficients in the model toward zero. Lasso has been found to perform well in many scenarios, although it was empirically demonstrated that it is outperformed by Ridge regression in nonsparse scenarios with highly correlated predictors (Tibshirani, 1996; Zou and Hastie, 2005). In such scenarios, *Elastic Net* regression (Zou and Hastie, 2005) has been found to improve the predictive power of Lasso by combining Lasso with the grouping effect of Ridge regression. The Elastic Net has two hyperparameters: α controlling the balance between L1 (lasso) and L2 (Ridge) penalties, and λ controlling the overall penalty strength. For the purpose of this paper, we use a readily available algorithm, GLMNET (Qian et al., 2013), for efficiently computing the Elastic Net problem. This is a coordinate descent algorithm for solving the following problem:

(13) $$\begin{array}{l} \underset{\text{W}}{\text{argmin}}\frac{1}{2N}∥\text{Y}-\text{X}\text{W}{∥}^{2}+\lambda \left[\left(1-\alpha \right)∥\text{W}{∥}^{2}/2+\alpha ∥\text{W}∥\right]. \end{array}$$

We used GLMNET for computing the Elastic Net solution for α = 0.25, α = 0.50, α = 0.75 and α = 1.00. We will henceforth refer the last case as the Lasso solution. The GLMNET has previously been used to estimate spectro-temporal receptive models (e.g., Willmore et al., 2016).

### 2.2. Evaluating performance

#### 2.2.1. Characterizing model fit

While the objective function of linear models is minimizing the mean-squared-error, the goodness of fit is typically analyzed in terms of Pearson's correlation between estimated and actual values for interpretability. The term *regression accuracy* will henceforth be used to characterize the goodness of fit for models trained and evaluated on attended audio features (*r*_*attended*_). For forward models, regression accuracies were measured by the Pearson's correlation between the actual EEG and the EEG predicted by the attended envelope over the test folds. This was done separately for each EEG channel. Similarly, for backward models, regression accuracies were measured by the correlation between the attended envelope and its EEG-based reconstruction. The regression accuracies were computed on test folds, using the nested cross-validation scheme described in section 2.2.3. This procedure ensures that the test data is not used during any part of the training process, including hyperparameter tuning. The regression accuracies were averaged over all test folds. Other metrics for assessing the predictive/reconstructive performance of the models have been previously proposed (Schoppe et al., 2016). However, for simplicity and to be consistent with previous studies (Ding and Simon, 2012a,b; O'Sullivan et al., 2015), this paper characterizes the goodness of the fit using Pearson's correlation coefficients.

In the forward case, the response at multiple EEG channels is predicted by the model. Rather than using multiple correlation coefficients to characterize the regression accuracy in this case, we chose to take the average of the correlation coefficients between the predicted channels and the actual EEG data as a validation score. We used the same metric over the test set to characterize the fit of the model. In the backward case, characterizing the fit is straightforward as the model predicts a single audio envelope that can be correlated with the attended audio envelope.

#### 2.2.2. Decoding selective auditory attention

Performance was also evaluated on a classification task based on the forward/backward stimulus-response model. The task of the classifier was to decide, on the basis of the recorded EEG and the two simultaneous speech streams presented to the listener (see section 2.4), to which stream the subject was attending. The classifier had to make this decision on the basis of a segment of test data, the duration of which was varied as a parameter (1, 3, 5, 7, 10, 15, 20, and 30 s), which will be referred to as the decoding segment length. This duration includes the kernel length of the forward/backward model (500 ms). The position of this segment of data was stepped in 1s increments throughout the evaluated data.

As described further in section 2.2.3, a nested cross-validation loop was used to tune the forward/backward stimulus-response model regularization parameter (where applicable) on training/validation data and test the trained classifier on unseen test data.

The classification relied on correlation coefficients between EEG and the attended speech, and between the EEG and the unattended speech. These correlation coefficients were computed over the aforementioned restricted time window. These coefficients were used to classify whether the subject was attending to one stream or the other. For a backward model, classification hinged merely on which correlation coefficient was largest (stream A or stream B). Performance of this classifier was evaluated on the test set. For a forward model, the situation is more complex because there is one model per EEG channel. For each of the 66 channels a pair of correlation coefficients was calculated (one each for unattended and attended streams), and this set of pairs was used to train a support vector machine (SVM) classifier with a linear kernel and a soft margin constant of 1. SVM classifiers were trained on the correlation coefficient features over the validation set that was used for hyperparameter tuning. The SVM classifier performance was finally evaluated on data from the held out test fold.

**Figure 1:**
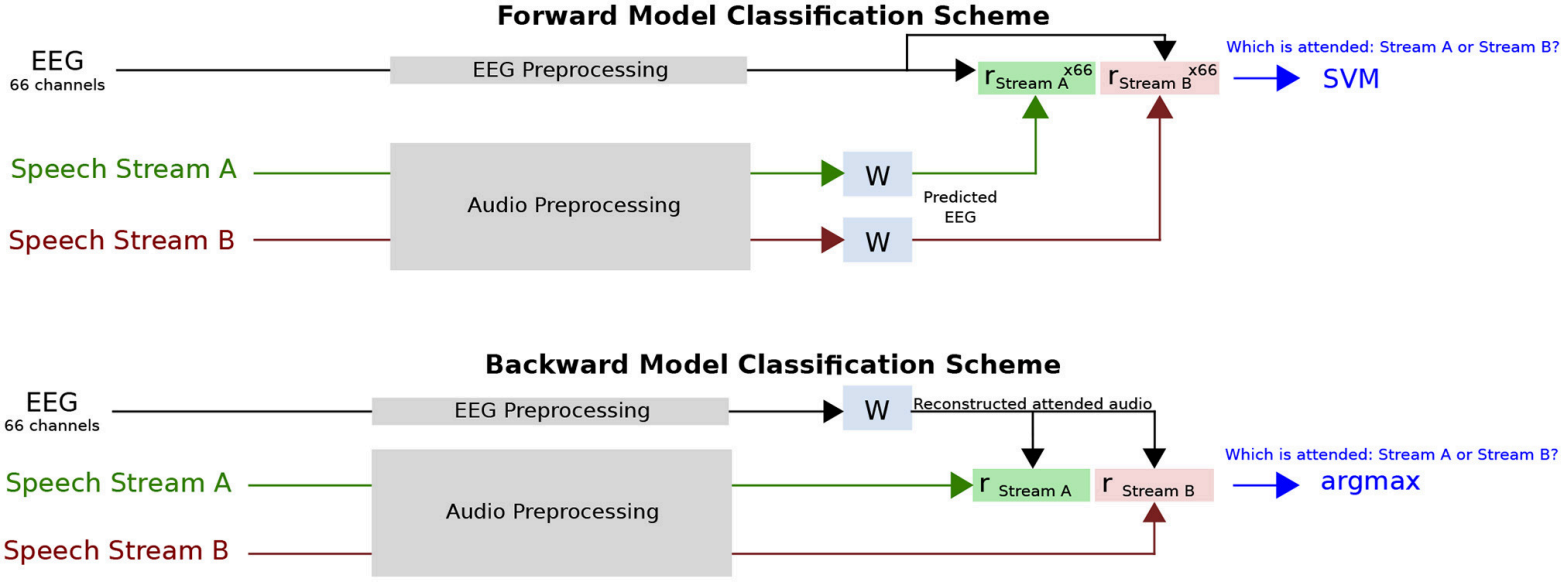
Diagram of classification task. For the forward model, 66 EEG channels are predicted from the speech stream A and B envelopes using the same linear mapping function, W. After correlation with the 66 channel EEG data, this results in 66 correlation coefficients for each speech stream, which are used as features for the SVM to distinguish the attended talker. For the backward model, a single attended audio envelope channel is estimated from the EEG data using the linear mapping function, W. After correlation with the speech stream A and B envelopes, a single correlation coefficient for each speech stream is obtained. Classification of the attended talker is performed by determining the larger coefficient.

The classifier score was averaged over all test folds. In every case, the classifier trained over the entire training/validation set was tested on a short interval of data, the duration of which was varied as a parameter, as explained above. An illustration of this classification task is shown in Figure 1.

Classification performance was characterized for different decoding segment durations using the raw classification score, receiver operating characteristic (ROC) curve, and information transfer rate (ITR). The raw classification score measured what proportion of trials were classified correctly. It should be noted that in measuring classification performance, the two classes were balanced. The ROC curve characterizes the true-positive and false-positive rates for decoding segment trials where the classifier discrimination function lies above a given threshold, as the threshold is varied. The classifier decision function is the distance between the classified point and the decision boundary, with the sign indicating the class label. In the case of an SVM classifier for the forward model, the decision function is a weighted sum of the input features (correlations), plus a bias term. In the case of the argmax function for the backward model, the decision function is the difference of the correlations between the reconstructed attended audio and the two speech streams. Thresholding the classifier discrimination function throughout the range of values it yields in a dataset affects the number of correctly and incorrectly classified trials (above threshold) out of the total number of correctly and incorrectly classified trials, which are the true and false positive rates, respectively.

The ITR metric corresponds to the number of classifications that can be reliably made by the system in a given amount of time. The dependency of ITR on decoding segment length is a tradeoff between two effects. On one hand, longer decoding segments allow more reliable decisions. On the other, short durations allow a larger number of independent decisions. There is thus an optimal decoding segment duration. A number of metrics to compute the ITR have been proposed. The most common is the Wolpaw ITR (Wolpaw and Ramoser, 1998), which is calculated in bits per minute as:

(14) $$\begin{array}{l} ITR_{W}=V\left[\log_{2}N+P\log_{2}P+\left(1-P\right)\log_{2}\frac{1-P}{N-1}\right], \end{array}$$

where *V* is the speed in trials per minute, *N* is the number of classes, and *P* is the classifier accuracy. We also report the Nykopp ITR, which assumes that a classification decision does not need to be made on every trial (Nykopp, 2001). This can be done by first calculating the confusion matrix *p* for classifier outputs where the classifier decision function magnitude exceeds a given threshold. Typically the larger the classifier decision function magnitude, the more accurate the classifier prediction. As such, raising the threshold on the decision function magnitude results in more accurate classifications at the expense of foregoing a classification decision on more trials. To obtain the Nykopp information transfer rate, the threshold on the classifier decision function magnitude is adjusted to maximize:

(15) $$\begin{array}{l} ITR_{N}=V[\underset{p\left(x\right)}{\max}\overset{N}{\underset{i=1}{\sum }}\overset{M}{\underset{j=1}{\sum }}p\left(w_{i}\right)p\left({ŵ}_{j}|w_{i}\right)\log_{2}p\left({ŵ}_{j}|w_{i}\right)\\ -\overset{M}{\underset{j=1}{\sum }}p\left({ŵ}_{j}\right)\log_{2}p\left({ŵ}_{j}\right)], \end{array}$$

where *p*(*w*_*i*_) is the probability of the actual class being class *i*, *p*(ŵ_*j*_|*w*_*i*_) is the probability of the predicted class being class *j* given the actual class being class *i*, and *p*(ŵ_*j*_) is the probability of the predicted class being class *j*. It is *p*(ŵ_*j*_|*w*_*i*_) and *p*(ŵ_*j*_) that are affected by decision function magnitude thresholding as this limits the number of trials on which a classification decision is made.

#### 2.2.3. Cross-validation procedure

The forward/backward stimulus-response models used in sections 2.2.1 and 2.2.2 were all trained and tested using cross-validation with a 10-fold testing procedure involving nested cross-validation loops. This procedure ensures that the test data used to evaluate the forward/backward model is not used during any part of the training process. During this cross-validation procedure the models were characterized under an N-fold testing framework where the data was divided into 10-folds. In this outer cross-validation loop, one fold was held out for testing (i.e., characterizing model fit and classifying the attended stream), while data from the remaining 9-folds were used to compute the forward/backward models using an inner cross-validation loop. This inner cross-validation loop was used to tune the hyperparameters. The stimulus-response models were in all cases fit to the envelope of the attended sound streams during the training phase. The regularization parameter was swept through a range of values to evaluate its effect on the correlation coefficient between the model prediction/reconstruction and the actual measured data for each inner cross-validation fold. For Ridge and Lasso regularization schemes that allowed a regularization parameter between zero and infinity, a parameter sweep was performed between 10^−6^ and 10^8^ in 54 logarithmically-spaced steps. This was done using the following formula:

(16) $$\begin{array}{l} \lambda_{n}=\lambda_{0}\times 1.848^{n},n\in \left[0,53\right], \end{array}$$

where $\lambda_{0}\equiv 10^{-6}$. For LRA, Elastic Net, and Shrinkage schemes, where the regularization parameter range was between 0 and 1, a parameter sweep was performed between 10^−6^ and 1 using a log-sigmoid transfer function that compresses the values between 0 and 1 using the following iterative formula:

(17) $$\begin{array}{l} \lambda_{n+1}=\text{logsig}\left(\text{ln}\left(\lambda_{n}\right)-\text{ln}\left(1-\lambda_{n}\right)+0.475\right),n\in \left[0,40\right]. \end{array}$$

The hyperparameter value that yielded the maximum correlation between the model prediction/reconstruction and actual measured data, averaged across all inner cross-validation folds, was used to evaluate the test set. Using this hyperparameter value, the weights of the models generated for each inner cross-validation fold were then averaged to generate an overall cross-validated model that could then be applied to the test set. It should be noted that for each test fold, the hyperparameter value was selected independently.

### 2.3. Implementation

The implementations of the forward/backward stimulus-response model algorithms used here are distributed as part of the Telluride Decoding Toolbox^2^, specifically in the FindTRF.m function of that toolbox. Data preprocessing, model training, and evaluation were implemented with the COCOHA Matlab Toolbox^3^.

### 2.4. Stimuli

A previous report gives a detailed description of the stimuli and data collection procedure (Fuglsang et al., 2017). This dataset is available online (Fuglsang et al., 2018). In brief, a set of speech stimuli were recorded by one male and one female professional Danish speakers speaking different fictional stories. These recordings were performed in an anechoic chamber at the Technical University of Denmark (DTU). The recording sampling rate was 48 kHz. Each recording was divided into 50-s long segments for a total of 65 segments.

### 2.5. Experimental procedure

The 50-s long speech segments were used to generate auditory scenes comprising a male and a female simultaneously speaking in anechoic or reverberant rooms. The two concurrent speech streams were normalized to have similar root-mean square values. The speech stimuli were delivered to the subjects via ER-2 insert earphones (Etymotic Research). The speech mixtures were presented binaurally to the listeners, with the two speech streams lateralized at respectively −60° and +60° along the azimuth direction and a source-receiver distance of 2.4 m. This was achieved using non-individualized head-related impulse responses that were simulated using the room acoustic modeling software, Odeon (version 13.02). Each subject undertook sixty trials in which they were presented the 50 s-long speech mixtures. Before each trial, the subjects were cued to listen selectively to one speech stream and ignore the other. After each trial, the subjects were asked a comprehension question related to the content of the attended speech stream. The position of the target streams as well as the gender of the target speaker were randomized across trials. Moreover, the type of acoustic room condition (either anechoic, mildly reverberant or highly reverberant) were pseudo-randomized over trials. In the analysis, data recorded from all acoustic conditions were pooled together. The reasons for doing this were twofold. Firstly, it provides sufficient data for the stimulus-response analysis. This is particularly important as insufficient data in worst case can lead to poorer model estimates (Mirkovic et al., 2016). Secondly, by using this approach we get a better idea of how well the models will generalize to different experimental conditions. This is an important practical aspect, as it gives a better estimate of how well a classifier will perform in different listening conditions (rather than just focusing on training on anechoic data and evaluating on anechoic data).

### 2.6. Data collection

Electroencephalography (EEG) data were recorded from 19 subjects in an electrically shielded room while they were listening to the stimuli described above. Data from one subject were excluded from the analysis due to missing data from several trials. The data were recorded using a Biosemi Active 2 system, with a sampling rate of 512 Hz. Sixty-four channel EEG data (10/20-system) were recorded from the scalp. Six additional electrodes were used for recording the EEG at the mastoids, and vertical and horizontal electrooculogram (V and H-EOG). Approximately 1 h of EEG data was recorded per subject. This study was carried out in accordance with the recommendations of “Fundamental and applied hearing research in people with and without hearing difficulties, Videnskabsetiske komitee.” The protocol was approved by the Science Ethics Committee for the Capital Region of Denmark. All subjects gave written informed consent in accordance with the Declaration of Helsinki.

### 2.7. Data preprocessing

#### 2.7.1. EEG data

50 Hz line noise and harmonics in the EEG data were filtered out by convolution with a $\frac{512}{50}$ sample square window (the non-integer window size was implemented by interpolation) (de Cheveigné and Arzounian, 2017). The EEG data was then downsampled to 64 Hz using a resampling method based on the Fast Fourier Transform (FFT). To downsample, this method reduces the size of the FFT of the signal by truncating high frequency components. An inverse FFT is then used to restore the signal to the time domain. A 1st order detrend was performed on the EEG data to minimize filter startup artifacts. EEG data were highpassed at 0.1 Hz using a 4th order forward-pass Butterworth filter. The group delay was less than 2 samples above 1 Hz.

The joint decorrelation framework (de Cheveigné and Parra, 2014) was employed to remove eye artifacts in an automated fashion. Let **X** = [*x*_*tj*_] be a matrix that contains EEG data from each electrode, *j*, for each time sample *t*. In this implementation, a conservative eye artifact time-point detection was first performed by computing a Z-score on 1–30 Hz bandpassed VEOG and HEOG bipolar channels and marking time samples where the absolute Z-score on either channel exceeded 4. This is similar to the eyeblink detection method implemented in the FieldTrip EEG processing toolbox (Oostenveld et al., 2011). This resulted in a subset of time samples, *A*, indexing the temporal locations of each EOG artifact. An artifact covariance matrix ${\text{R}}_{A}={\text{X}}_{A}^{T}{\text{X}}_{A}$ was then computed from the EEG (and EOG) data, **X**_*A*_ = [*x*_*aj*_], at the artifact time samples *a*∈*A*. After using principal component analysis to whiten **R**_*A*_ and **R**, the generalized eigenvalue problem was then solved for **R**_*A*_**v** = λ**Rv**, where **R** = **X**^*T*^**X** is the covariance matrix for the entire EEG dataset. The resulting eigenvectors **V**, sorted by eigenvalue, explain the maximum difference in variance between the artifact and data covariance matrices. Components corresponding to eigenvalues > 80% of the maximum eigenvalue were regressed out of the data. In practice, this 80% threshold is a conservative one, typically resulting in the removal of one or two components. Lastly, the EOG channels were removed from the data, which was then referenced to a common average over all channels.

For the forward/backward model analysis, the EEG was bandpassed between 1–9 Hz using a windowed sync type I linear-phase finite-impulse response (FIR) filter, shifted by its group delay to produce a zero-phase (Widmann et al., 2015) with a conservatively chosen order of 128 in order to minimize ringing effects. This frequency range was selected as it has been shown that cortical responses time-lock to speech envelopes in this range (O'Sullivan et al., 2015). As part of the cross-validation procedure, individual EEG channels were finally centered and standardized (Z-normalized) across the time dimension using the individual channel mean and standard deviation of the training data. A kernel length of 0.5 s (33 samples) was used when computing the forward/backward models.

#### 2.7.2. Audio features

The forward/backward stimulus-response model estimation methods used for attention decoding attempt to characterize a relationship between features of attended speech streams and EEG activity. We calculated temporal envelope representations from each of the clean speech streams (i.e., without reverberation). We did not try to derive them from the reverberant or mixed audio data, as explored elsewhere (Aroudi and Doclo, 2017; Fuglsang et al., 2017). In trials with reverberant speech mixtures, we used envelope representations of the underlying clean signals to estimate the models. To derive the envelope representations, we passed monaural versions of both attended and unattended speech streams through a 31-band gammatone filterbank with a frequency range of 80–8,000 Hz (Patterson et al., 1987). The envelope of each filterbank output was calculated via the analytic signal obtained with the Hilbert transform, raised to the power of 0.3. This rectification and compression step was intended to partially mimic that which is seen in the human auditory system (Plack et al., 2008). The audio envelope was then calculated by summing the rectified and compressed filterbank outputs across channels. The audio envelope data was subsequently downsampled to the same sampling frequency as the EEG (64 Hz) using an FFT-based resampling method. The EEG and envelopes were then temporally aligned using start-trigger events recorded in the EEG. The envelopes were subsequently lowpassed at 9 Hz. As part of the cross-validation procedure, audio envelopes were finally centered and standardized (Z-normalized) across the time dimension using the mean and standard deviation of the attended speech envelope in the training data.

### 2.8. Statistical analysis

All statistical analyses were calculated using MATLAB. Repeated-measures analysis of variance (ANOVA) tests were used to assess differences between the regression accuracies (section 2.2.1) and classification performances section 2.2.2 obtained with the different forward/backward model estimation methods. Regression accuracies and classification performances for individual subjects were averaged across folds prior to statistical comparison.

Given the non-Gaussian distribution of regression accuracies (range -1 to 1) and classification performance metrics (range 0 to 1), Fisher Z-transforms and arcsine transforms were applied to these measures, respectively, prior to statistical tests and correlations.

## 3. Results

The forward/backward stimulus-response model estimation methods introduced in section 2 were used to decode attended speech envelopes from low-frequency EEG activity. The following sections analyze results with metrics of (1) regression accuracy, (2) classification accuracy, (3) receiver operating characteristic (ROC), and (4) information transfer rate (ITR). Results are shown for each of the regularization schemes, for both forward and backward models. For each regularization scheme, the regularization parameter(s) are tuned to maximize regression accuracy. These parameter values are then used for all regression and classification comparisons. Regression accuracy compares different regularization schemes in predicting/reconstructing test data using the optimal regularization parameter. Classification accuracy uses the regression accuracy values to classify the attended/unattended talker and compares the different regularization schemes in performing this task. The ROC curve visualizes the relationship between the true and false-positive rates for different classifier discrimination function thresholds. Lastly, the ITR describes the impact of decoding segment length on the bit-rate, for different points on the ROC curve.

### 3.1. Regularization parameter tuning

The forward/backward model estimation methods, except for the OLS method, use regularization techniques to prevent overfitting and therefore require a selection of the appropriate tuning parameters. Figure A1 in Supplementary Material shows the correlation coefficient between estimated (validation set) data and the actual target data (*regression accuracy*) over a range of regularization parameters. In general, there is a broad region where validation regression accuracy is flat, which peaks before quickly falling off with increasing λ. It is also apparent that the regression accuracies obtained with backward models generally are higher than those obtained with forward models.

Figure A2 in Supplementary Material shows regression accuracies for forward/backward models with Elastic Net penalties. Unlike the other linear models investigated in the present study the Elastic Net has two hyperparameters. The α parameter adjusts the balance between *L*1 and *L*2 penalties. Similar to the other regularization schemes, for each value of α, there is a broad range of λ values that give good correlation performance.

### 3.2. Regression accuracy

**Figure 2:**
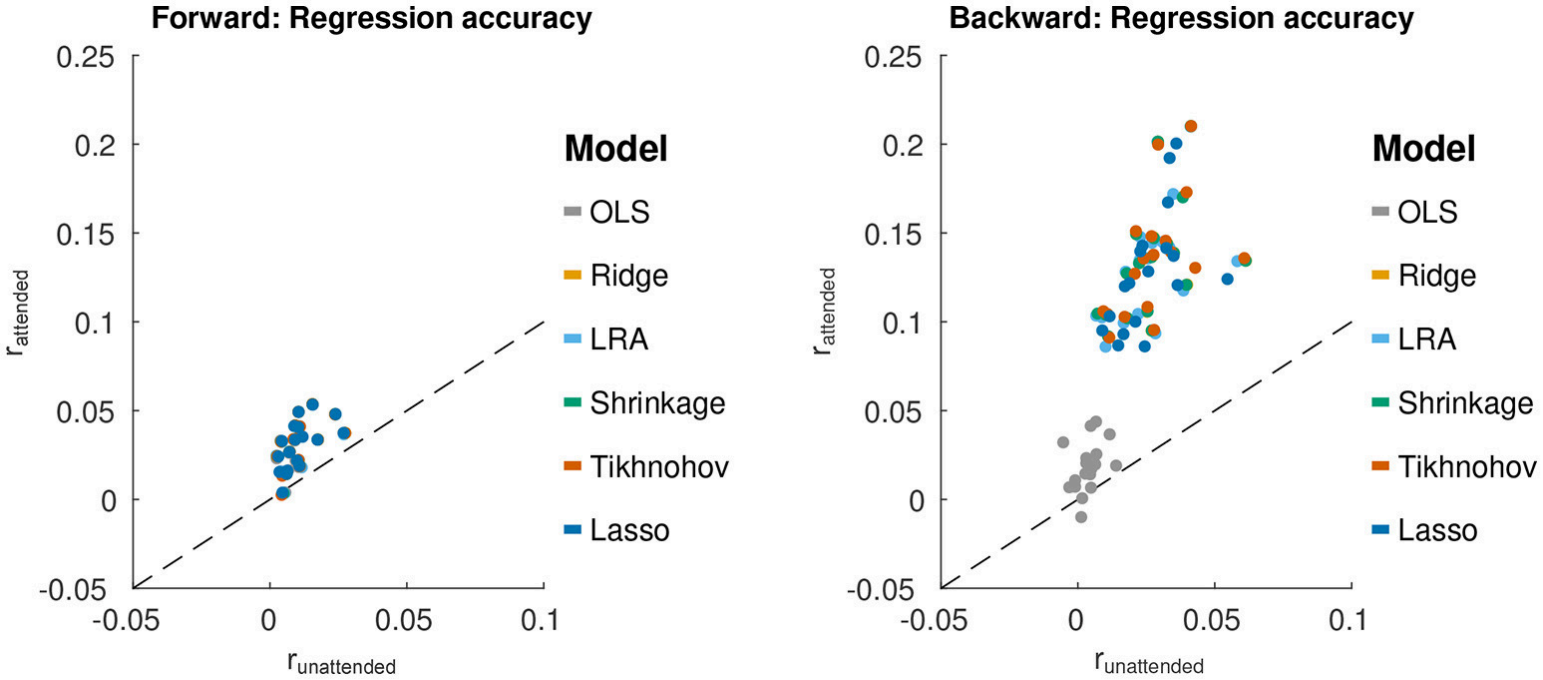
Test set regression accuracies (*r*_*attend*_) for each forward/backward model estimation method plotted against *r*_*unattend*_. **(Left)** Results from the forward modeling approach. Points for each regularization scheme are close to each other, and thus appear to fall on top of each other. **(Right)** Results from the backward modeling approach. For each scheme (represented by a color), each point represents average data from one subject. The black line shows *r*_*attend*_ = *r*_*unattend*_.

For each regression method (and each value of α for Elastic Net), the forward/backward stimulus-response model was estimated and the optimum lambda estimated on the training/validation set. This optimal model was then applied to the test set, and the regression accuracy was compared between regression methods. This is shown in Figure 2. One might expect that the averaging of prediction-response correlations across channels for the forward model may have resulted in lower regression accuracies compared to the backward model. This was demonstrating using a *t*-test between the forward and backward models, over all regularization schemes and subjects [Δ = 0.083, *T*_(107)_ = 17.8, *p* = 1.1 × 10^−33^]. However, when using maximum correlation across channels, instead of the average, for the forward model, there was still a significant difference [Δ = 0.045, *T*_(107)_ = 9.8, *p* = 9.4 × 10^−17^].

For forward models, a repeated measures ANOVA with regularization method as the factor found no significant effect of regularization method on the average of correlation coefficients, even when using the average of the correlation coefficients of the 5 channels with the largest correlation coefficients for each subject. For the backward models, a similar repeated measures ANOVA, found a significant effect of regularization method on regression accuracy [*F*_(5, 85)_ = 78.0, *p* < 1.0 × 10^−16^]. Tikhonov regularization yielded a regression accuracy that was significantly greater than each of the other schemes, using a Bonferonni correction to account for the family-wise error rate (*p* < 0.045). This is contrary to the expectation that Ridge regression would outperform Tikhonov for the backward model due to the inter-channel leakage introduced by the Tikhonov kernel. Moreover, OLS had a regression accuracy that was significantly smaller than the other schemes (with Bonferonni correction, *p* < 1.3 × 10^−10^). This highlights the importance of regularization for the backward models.

For Elastic Net regularization, α values was characterized at 0.25, 0.5, 0.75, and 1 (Lasso) to sample different degrees of sparsity/smoothness. The value α = 0 (Ridge) was not sampled due to sub-optimal solver performance near this point. A repeated measures ANOVA analysis with factors of α and subject, using optimal λ values, showed no significant effect of α for forward models. This means that adjusting the model sparsity had no significant effect on the regression accuracy. However, a significant effect of α was found for backward models [*F*_(3, 51)_ = 12.4, *p* = 3.3 × 10^−6^]. A *post-hoc* paired *t*-test with a Bonferonni correction revealed that the best regression accuracy was obtained with α = 0.25 (*p* = 6.2 × 10^−4^). It was, however, noted that the average difference between regression accuracies for α = 0.25 and α = 1 was only 8 × 10^−4^.

To obtain an estimate of the significance of the regression accuracies presented in Figure 2, we randomized the phase of the audio data passed to the forward models, and the phase of the EEG data passed to the backward models. The goal was to provide an estimate of the correlation noise floor for the models. The models were those trained on unaltered data using each of the regularization schemes. Randomizations were performed 100 times per subject to yield an estimate of the noise floor regression accuracies. The regression accuracies were computed the same way as before. A two-sample Kolmogorov-Smirnov test conducted pairwise showed that, within subjects, the distribution of noise floor correlations were not significantly different between regularization schemes, or channels in the case of the forward model. The within-subject distributions were thus combined, and a two-sample Kolmogorov-Smirnov test was performed pairwise between subjects. No significant difference in distributions was found between subjects. As such, all distributions were combined. The 95% confidence interval of the noise floor correlations was [-0.001, 0.001] for the forward model and [-0.032, 0.032] for the backward model.

### 3.3. Classification accuracy

**Figure 3:**
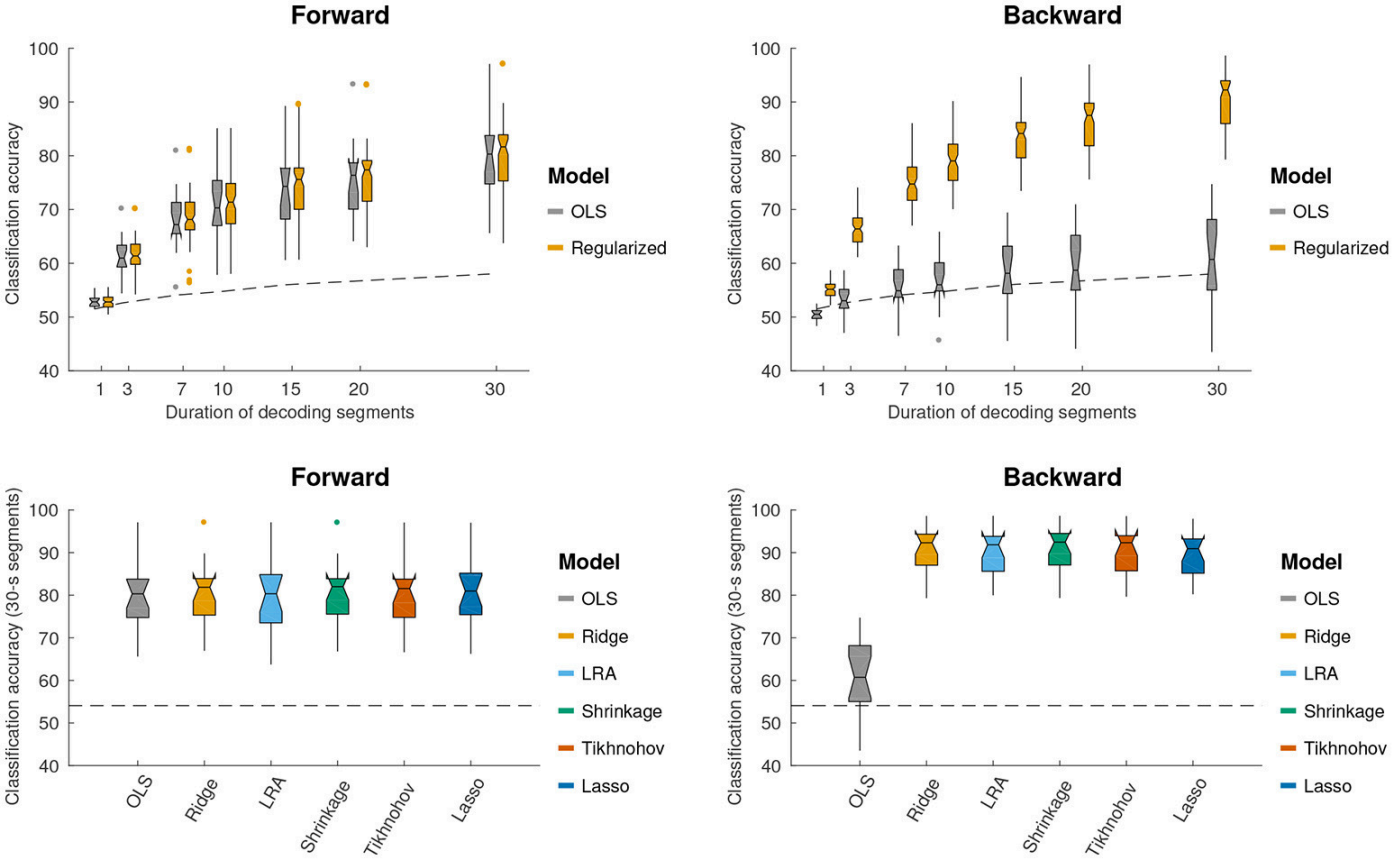
Using different forward/backward models to decode selective auditory attention from multi-channel EEG data. Classification performance is shown for different decoding segment lengths (1, 3, 7, 10, 15, 20, 30 s). (**Top**, left and right) Show the classification performance for forward and backward models respectively. Performance is shown for the OLS scheme and an average across regularized schemes. Regularized schemes were averaged to concisely illustrate the higher classification accuracy obtained by these schemes compared to OLS for the backward model, but not the forward model. (**Bottom**, left and right) Show the classification performance for 30 s long decoding segments. The different regularization schemes are shown in different colors (see legend). Notched boxplots show median, and first and third quartiles. Whiskers show 1.5 × IQR. Dots indicate outliers. The dashed line shows the above-chance significance threshold at *p* = 0.05.

We further sought to investigate how the different forward/backward models perform in terms of discriminating between attended and unattended speech on a limited segment of data. The duration of the segment was varied as a parameter (1, 3, 5, 7, 10, 15, 20, and 30 s). This was characterized on held-out test data for each TRF method, using the λ value that yielded the maximum regression accuracy in the validation data. The results from this analysis are shown in Figure 3. A 2-way repeated measures ANOVA with factors of regularization scheme and model (forward or backward), based on 30 s decoding segment lengths, found a main significant difference between backward and forward models [*F*_(1, 17)_ = 17.3, *p* = 6.5 × 10^−4^], with a significant interaction with the effect of regularization scheme [*F*_(5, 85)_ = 208.9, *p* < 1.0 × 10^−16^]. A *post-hoc* paired *t*-test showed that backward model performs better than the forward model for all regularization schemes excluding the case where ordinary least squares (OLS) was applied [*T*_(17)_ = 9.35, *p* = 4.2 × 10^−8^]. For OLS, the forward model outperformed the backward model [*T*_(17)_ = 7.32, *p* = 1.2 × 10^−6^].

The interaction of the effect of regularization scheme on the classification accuracy of forward and backward models was investigated. A repeated measures ANOVA with factors of regularization scheme, applied only to the forward TRF classification accuracy scores, found no significant effect of regularization scheme on classification accuracy. This is consistent with the lack of significant differences being detected in regression accuracies for different forward model regularization schemes, even when limiting the number of channels to 5 with the highest regression accuracies. In this case, the SVM classifier can be viewed as a data-driven approach to select channels that are most relevant to attention classification. For the backward models, however, a significant effect of regularization scheme on classification accuracy was found [*F*_(5, 85)_ = 229.4, *p* < 1.0 × 10^−16^]. A *post-hoc* paired *t*-test analysis with a Bonferonni correction revealed that the classification accuracy for the OLS scheme was significantly worse than each of the others ($\bar{\Delta }=-29.1$, *p* < 7.9 × 10^−10^). Lasso performed significantly worse than each of the remaining schemes ($\bar{\Delta }=-1.2$, *p* < 0.040). In short, regularized backward schemes outperform OLS by a relatively large margin, as seen in Figure 3.

For Elastic Net regularization, a repeated measures ANOVA with factors of α and subject did not find any significant effect of α on classification accuracy for forward or backward models.

In summary, for the forward model there was no difference between schemes (regularization and OLS), and for the backward model there was no difference between Ridge, Tikhonov, Shrinkage and LRA, but all regression methods were better than OLS.

#### 3.3.1. Relation to regression accuracy

**Figure 4:**
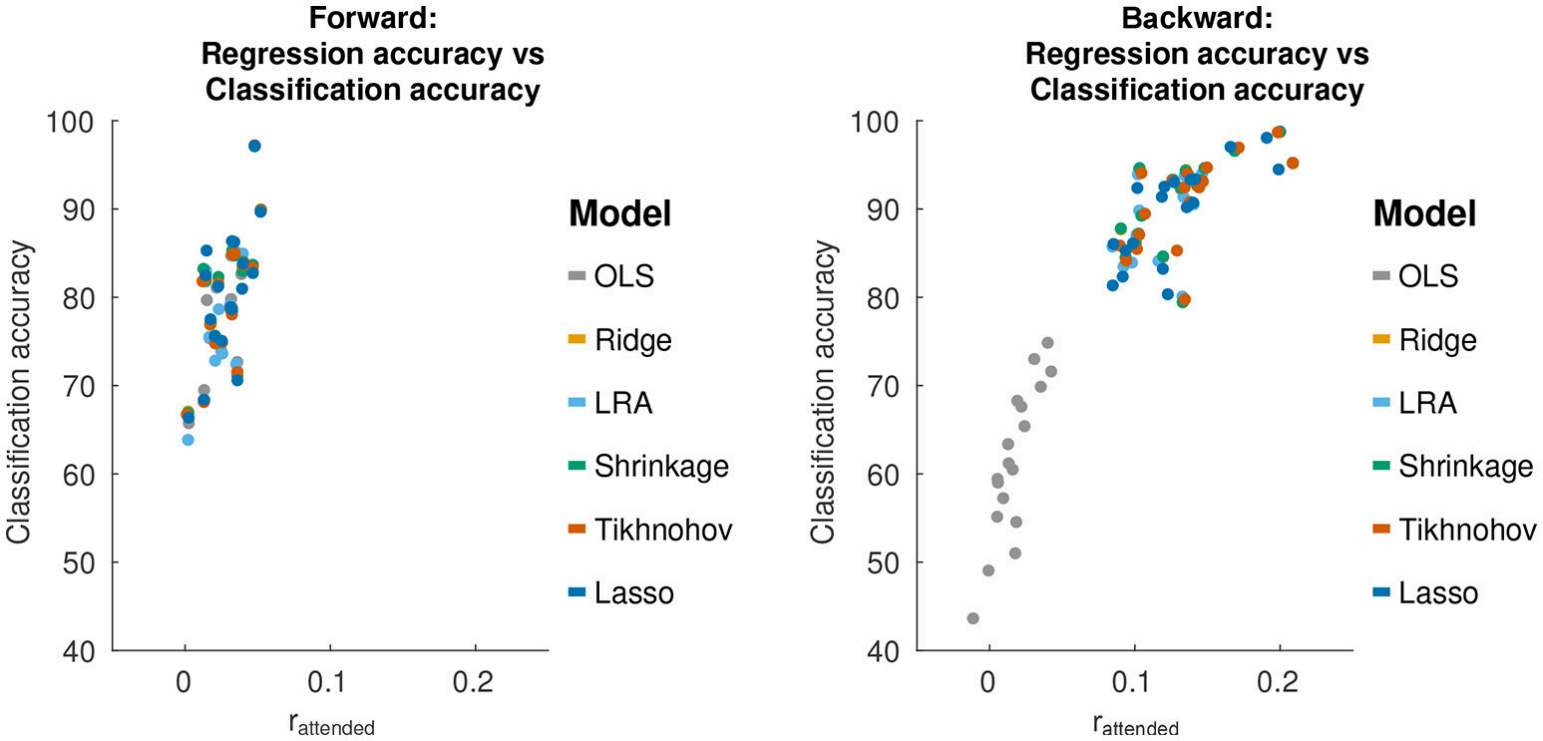
Relationship between regression accuracy and classification accuracy, using 30 s decoding segment lengths.

The discrimination between attended and unattended speech streams from EEG data is done in two stages: the computation of regression accuracies, followed by classification. We sought to investigate how the classification accuracies obtained with each model relate to the test set regression accuracies. A plot of this relationship is shown in Figure 4.

For forward models, the average correlation between regression accuracy and classification performance across decoding segments and over all regularization schemes is 0.69 [*T*_(108)_ = 9.83, *p* = 2.2 × 10^−16^]. For backward models, the correlation between the regression accuracy and classification performance is 0.89 [*T*_(108)_ = 22.4, *p* < 1.0 × 10^−16^]. This suggests that classification performance varies with regression accuracy. However, as was previously described for the backward models, while Tikhonov regularization achieved a significantly higher regression accuracy compared to all other methods, it did not achieve a significantly higher classification performance compared to Shrinkage, Ridge Regression or LRA. To explain this, we examined the classification feature in terms of the difference between class means (${\bar{r}}_{attend}-{\bar{r}}_{unattend}$) and the within-class standard deviation ($\sqrt{0.5\left(\sigma_{r_{attend}}^{2}+\sigma_{r_{unattend}}^{2}\right)}$). Both of these terms affect the separability between classes.

For backward models, Tikhonov regularization had a significantly larger difference between class means compared to Ridge Regression and Shrinkage [Tikhonov>Ridge: *T*_(17)_ = 2.62, *p* = 0.018], [Tikhonov>Shrinkage: *T*_(17)_ = 2.59, *p* = 0.019]. At the same time, the between-class standard deviation was also significantly larger for Tikhonov regularization [Tikhonov>F_(100, 100)_ = 2.37, *p* = 1.2 × 10^−5^], [Tikhonov>Shrinkage: *F*_(100, 100)_ = 2.37, 1.4 × 10^−5^]. This suggests that while Tikhonov regularization yields a better regression accuracy (correlation coefficient), this is offset by an increased variance in the regression accuracy computed over short decoding segments, nullifying any potential gains in classification performance.

### 3.4. Receiver operating characteristic

**Figure 5:**
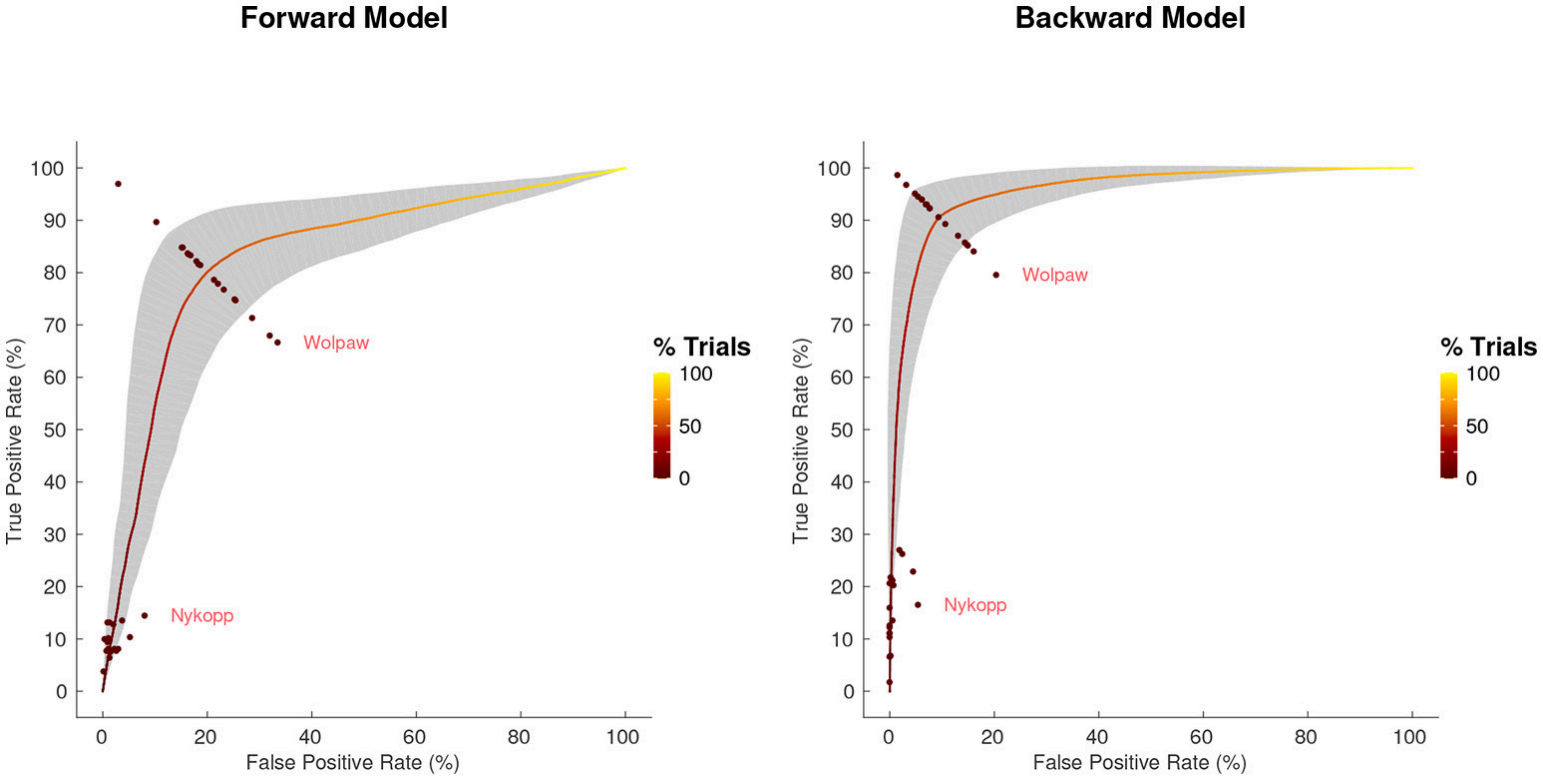
Average receiver operating characteristic curve, with standard deviation band, for 30 s decoding segments using Tikhonov regularization. Points at which Wolpaw and Nykopp information transfer rates were evaluated for each subject are shown. Color along curve indicates percentage of decoding segment trials evaluated to obtain each point. The gray band indicates the standard deviation boundaries of the curve in both x and y directions.

The receiver operating characteristic (ROC) curve, shown in Figure 5, shows the relationship between the true-positive rate and false-positive rate for decoding segment trials where the classifier discrimination function lies above a given threshold, as the threshold is varied. The classification accuracy score that we report corresponds to the point on the ROC that lies along the line between (0,100) and (100,0). This is also the point at which the Wolpaw information transfer rate (ITR) is estimated, whereas the Nykopp ITR estimation finds a point that lies further left along the ROC curve. The area under the curve is highly correlated with classification accuracy (over all regularization schemes and decoding segment lengths, [*r* = 0.99, *T*_(862)_ = 219.9, *p* < 1.0 × 10^−16^]. The Nykopp ITR, on the other hand lies further left along the ROC curve, demonstrating that by avoiding the classification of some trials, it is possible to maximize the ITR.

### 3.5. Information transfer rate

**Figure 6:**
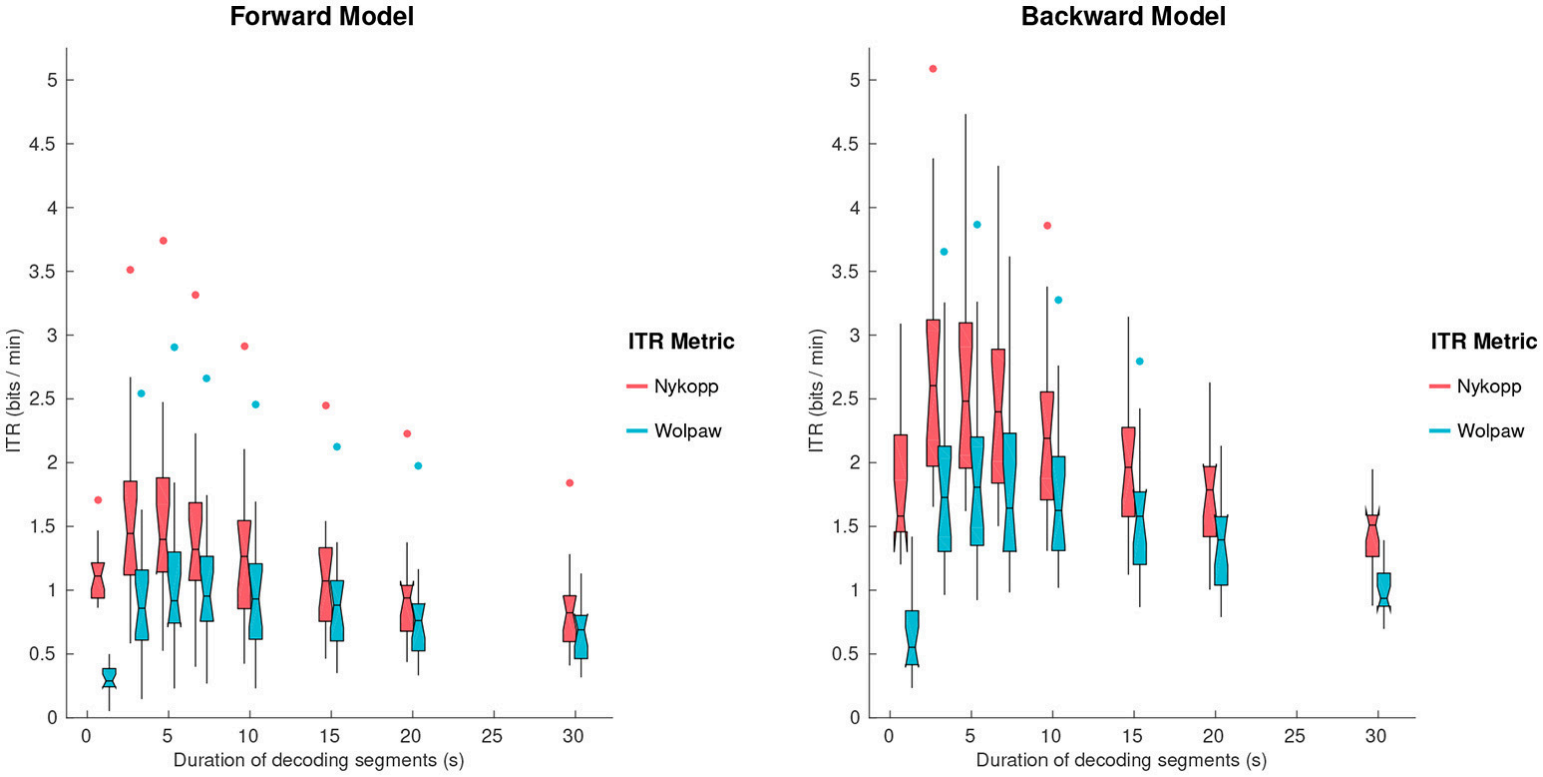
Wolpaw and Nykopp information transfer rates (ITR) as a function of decoding segment duration for the forward and backward models, using Tikhonov regularization. Notched boxplots show median, and first and third quartiles. Whiskers show 1.5 × IQR. Dots indicate outliers.

The Wolpaw ITR represents the transfer rate when all decoding segments are classified, whereas the Nykopp ITR represents the maximum achievable transfer rate when some classifications are withheld based on classification discrimination function output. Figure 6 shows the Wolpaw and Nykopp ITR values as a function of decoding segment duration, based on models computed with Tikhonov regularization. Both the Wolpaw and Nykopp ITR show an increase followed by a decrease with increasing decoding segment duration. The plots suggest that for brain computer interface applications with fixed decoding segment lengths, it may be advisable to use decoding segments of 3–5 s to maximize the ITR. While the Nykopp measure is an upper-bound, its increase over the Wolpaw ITR value [forward model, 5 s: *T*_(17)_ = 13.1, *p* = 1.3 × 10^−10^], [backward model, 5 s: *T*_(17)_ = 16.7, *p* = 2.7 × 10^−12^] demonstrates that by adjusting the classifier decision function cutoff, it could be possible to increase the ITR.

## 4. Discussion

In this study, we systematically investigated the effects of forward/backward stimulus-response model estimation methods on the ability to decode and classify attended speech envelopes from single-trial EEG responses to speech mixtures. The performance of stimulus/EEG decoders based on forward models (mapping from attended speech envelopes to multi-channel EEG responses) and backward models (mapping from EEG response back to speech envelopes) were compared. It was found that the backward models outperformed the forward models in terms of regression and classification accuracies. While forward models could be expected to have higher regression accuracies due to the averaging of correlation coefficients across channels for forward models, the regression accuracy for the backward model was still higher when compared to the maximum correlation coefficient across channels for the forward model. We hypothesize that the models do a better job of reconstructing audio (the backward model) than predicting EEG data (the forward model) because the EEG data contains a lot of information from other brain functions. It is impossible to predict these signals from the stimulus, hence the limited success of a forward model, but it is possible to filter them out, hence the better performance of a backward model. There are also other fundamental differences between the models, such as statistical and structural properties of the regressor variable, and number of parameters estimated. For instance, the eigenspectrum of the EEG autocovariance matrix in Figure A3 in Supplementary Material suggests that the matrix is ill-conditioned, particularly compared to that of the speech envelope. Different regularization schemes were not found to significantly affect the forward model classification accuracies. However, for the backward models, the decoding schemes that yielded the best classification accuracy were Ridge Regression, LRA, Shrinkage and Tikhonov. Lasso had a lower classification accuracy by a small but significant margin. Classification accuracy increased monotonically as a function of duration, reflecting the greater amount of discriminative information available in longer segments. ITR however peaked at an intermediate segment duration, reflecting the tradeoff between the accuracy of individual classification judgments (greater at long durations) and number of judgments (greater at short durations). The optimum was around 3–5 s.

For the analysis, we used different linear approaches to decode selective auditory attention from stimulus and EEG data. These analyses all relied on the explicit assumption that the human cortical activity selectively tracks attended and unattended speech envelopes. To fit the models, we made a number of choices based on common practices in literature, and with the goal of being able to compare forward/backward models and regularization schemes. For example, a 500 ms kernel was used as was done by others (Fuglsang et al., 2017). While shorter kernels have been explored as well (O'Sullivan et al., 2015), a longer one tests the ability of the model estimation method to handle a larger dimensionality and allows for a more flexible stimulus-response modeling capturing both early and late attentional modulations of the neural response. Additionally, we chose to focus on 1–9 Hz EEG activity as the attentional modulation of EEG data has been found prominent in this range. It is likely that other neural frequency bands robustly track attended speech (e.g., high gamma power Pasley et al., 2012) and that the neural decoders potentially could benefit from having access to other neural frequency bands. This is, however, outside the scope of this paper.

### 4.1. Decoding selective auditory attention with forward and backward models

The forward models performed significantly worse than the backward models in terms of classification accuracies. Single-trial scalp EEG signals are inherently noisy, in part because activity picked up by each electrode reflects a superposition of activity from signals that are not related to the selective speech processing (Blankertz et al., 2011). We refer here to any aspects of the EEG signals that systematically synchronize with the attended speech streams as target signals and anything that does not as noise. To improve the signal-to noise ratio one can efficiently use spatio-temporal filtering techniques. This in part relates to the fact that stimulus-irrelevant neural activity tends to be spatially correlated across electrodes. The spatio-temporal backward models implicitly exploit these redundancies to effectively filter out noise and improve signal-to-noise-ratio. This makes them fairly robust to spatially correlated artifact activity (e.g., electro-ocular and muscle artifacts) when trained on data from a large number of electrodes. This is also reflected in the high classification accuracies that were obtained with the backward models. However, for the relatively high number of electrodes used in this study, it was found that the spatio-temporal reconstruction filters were effective only when properly regularized.

The forward models, on the other hand, attempt to predict the neural responses of each electrode in a mass-univariate approach. These models do not, therefore, explicitly use cross-channel information to regress out stimulus-irrelevant activity. The relative contribution of the individual channels to the classification accuracies were instead found via an SVM trained on correlation coefficients computed per channel, over short time segments. In short, backward models remove spatial information prior to classification when regressing out non-stimulus-related activity, whereas forward models preserve this information, but do not regress out non-stimulus-related activity. It can therefore be beneficial to apply dimensionality reduction techniques [e.g., independent component analysis (Bell and Sejnowski, 1995) or joint decorrelation (de Cheveigné and Parra, 2014)] to represent the EEG data as a linear combination of fewer latent components prior to fitting the forward models. Alternatively, canonical component analysis can be used to jointly derive spatio-temporal filters for both audio and EEG such that the correlation between the filtered data is maximized (de Cheveigné et al., 2018).

#### 4.1.1. Regularization

Each regularization scheme makes certain assumptions and simplifications that are therefore adopted by studies employing them. Because these methods have not been previously evaluated side by side, it is unknown how valid these assumptions are.

While no regularization (OLS) was found to work well for forward models in producing classification accuracies roughly in line with regularized models, this method performs relatively poorly when applied to backward models. This is likely reflective of the higher dimensional kernel required for the backward problem. For comparison, a forward model had 33 parameters (per channel) that needed to be fit, whereas a backward model had 2,178 parameters.

We generally found that the reconstruction accuracies (*r*_*attend*_) plateaued over a large range of λ values for linear models (Figure A1).

Elastic net regularization permits the adjustment of the balance between L1 and L2 regularization via the α parameter. For the backward model, it was shown that a smaller α value improved the correlation between the reconstructed and attended audio stream by only a narrow margin.

The α value had no significant impact on classification accuracy for either forward or backward models. As such, the higher classification performance of Ridge Regression (α = 0), compared to Lasso (α = 1) may be a result of differences between the closed form solution used for Ridge Regression and the coordinate descent solution used for the Elastic Net, as well as between the solvers themselves (MATLAB's *mldivide* vs. GLMNET, Qian et al., 2013).

Another coordinate descent method, known as boosting, has been used in several studies (David et al., 2007; Calabrese et al., 2011; Thorson et al., 2015). It has been shown that boosting promotes sparse solutions in the context of spectro-temporal receptive fields with single-unit recordings (David et al., 2007). This method was not explored in the present study because boosting tends to be computationally intractable for backward models due to the high number of parameters, and because it involves a large set of hyperparameters. This makes a direct comparison of the regularization methods difficult. Instead we used the Elastic Net algorithm to investigate how the stimulus-response models could benefit from sparsity.

For the forward model, all regularization schemes yielded regression and classification accuracies that were not significantly different from each other. For the backward model, Tikhonov regularization yielded the best regression accuracy, despite the fact that cross-channel leakage may have lead to a suboptimal solution. However, it was found that the improved regression accuracy did not lead to a better classification accuracy compared to other regression schemes with closed-form solutions (i.e., Ridge, Shrinkage, and LRA) due to an associated increased variance in the correlation coefficient computed over short decoding segment lengths. It has been reported that, in practice, the Ridge Regression approach appears to perform better than LRA (Vajargah, 2013); however, no significant difference was found in the present study. LRA removes lower variance components after the eigendecomposition of **X**^*T*^**X**, essentially performing a hard-threshold. In contrast, Ridge Regression is a smooth down-weighting of lower-variance components (Blankertz et al., 2011).

### 4.2. Realtime performance

The information transfer rate results provide insight into how classification performance can be optimized. It is worth noting that the ITR measures represent particular points along the ROC curve, as is illustrated in Figure 5. For a binary classification problem, with balanced classes, the Wolpaw ITR corresponds to the point on the ROC curve along the line connecting the corners of the plot at coordinates (100,0) and (0,100). The Nykopp ITR, on the other hand corresponds to the point that maximizes the ITR, essentially trading the number of classified samples for increased classification accuracy. In practice, other considerations besides ITR can influence the choice of the point on the ROC. For instance, if there is a high penalty on incorrect classifications, then the classifier threshold may be adjusted to operate at another point on the ROC curve. In short, the ROC and ITR are useful tools in identifying a suitable balance between sensitivity and specificity.

The ITR results in the present study suggest a 3–5 s decoding segment length to achieve the maximum bit-rate. It should be noted that this assumes that switches in attention can occur frequently, on the order of the decoding segment length, such as in a real-world cognitive control setting where system response latency is an important constraint. In cases, where switches in attention are known to be sparse *a priori*, it may instead be more desirable to increase decoding segment length and sacrifice bit-rate to put more emphasis on accuracy, since the loss in bit rate due to long decoding segments is only evident during attention switches. Such an approach was taken by O'Sullivan et al. (2017), where the theoretical performance of a realtime backward model decoding system was characterized for switches in attention every 60 s. In that study, a decoding segment length between 15 and 20 s was reported as optimal to achieve the best speed-accuracy tradeoff.

### 4.3. Summary

There are many methods that can be used to compute forward/backward stimulus-response models. The present study uses a baseline dataset and procedures for the evaluation of these methods. In consideration of the multiple applications in which forward/backward models are used, primarily dealing with reconstruction accuracies or classification performance, this paper considered multiple metrics of performance. By characterizing the regularization and performance of the model estimation methods, and the relationship between performance metrics, a more complete understanding of the validity of the assumptions underlying each method is provided, as well as the impact of the assumptions on the end result. While these experiments were done with EEG data, we expect that the results apply equally to magnetoencephalography (MEG) data. The key findings from this study were (1) the importance of regularization for the backward model, (2) the superior performance of Tikhonov regularization in achieving higher regression accuracy although this does not necessarily entail superior classification performance, and (3) optimal ITR can be achieved in the 3–5 s range and by adjusting the classifier discrimination function threshold.

## Author contributions

DW, SF, JH, EC, MS, and AdC contributed to the code used in the paper. DW, SF, JH, and AdC determined the data analysis procedure. DW created some of the figures, performed statistical analyses, wrote parts of the paper, and was responsible for the overall paper. SF created some of the figures, and wrote parts of the paper. JH, MS, and AdC provided critical feedback on the paper.

### Conflict of interest statement

MS was employed by Google. The remaining authors declare that the research was conducted in the absence of any commercial or financial relationships that could be construed as a potential conflict of interest.

## Footnotes

^1^http://doi.org/10.5281/zenodo.1199011

^2^http://www.ine-web.org/software/decoding

^3^http://doi.org/10.5281/zenodo.1198430
