# Supplementary material for: A Comparison of Regularization Methods in Forward and Backward Models for Auditory Attention Decoding
Source: Front Neurosci. 2018 Aug 7;12:531. doi: 10.3389/fnins.2018.00531 (PMC6090837; doi:10.3389/fnins.2018.00531)
Supplement: Supplementary file 1 [file Data_Sheet_1.pdf]

APPENDIX

The effect of regularization parameters on regression accuracy are shown in figures A1 and A2. The eigenspectra of the audio and EEG autocovariance matrices are shown in figure A3.

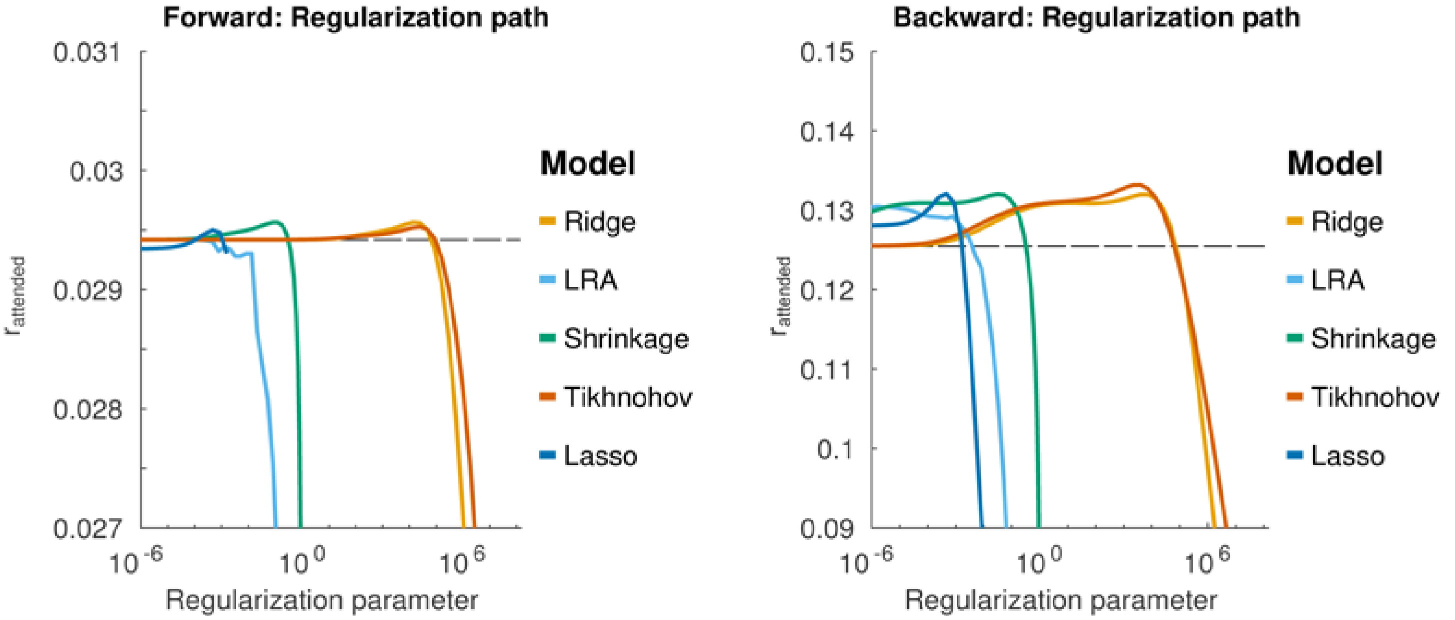

Figure A1. Group-mean validation-set regression accuracies obtained with different forward/backward model estimation methods as the regularization parameters λ are varied. The left-hand and right-hand panel present results obtained with forward and backward models, respectively. The x axis shows the strength of the λ regularization parameters. The y axis shows the regression accuracies in terms of Pearson's correlation coefficients between estimated data and target data. The dashed line shows the regression accuracy for OLS.

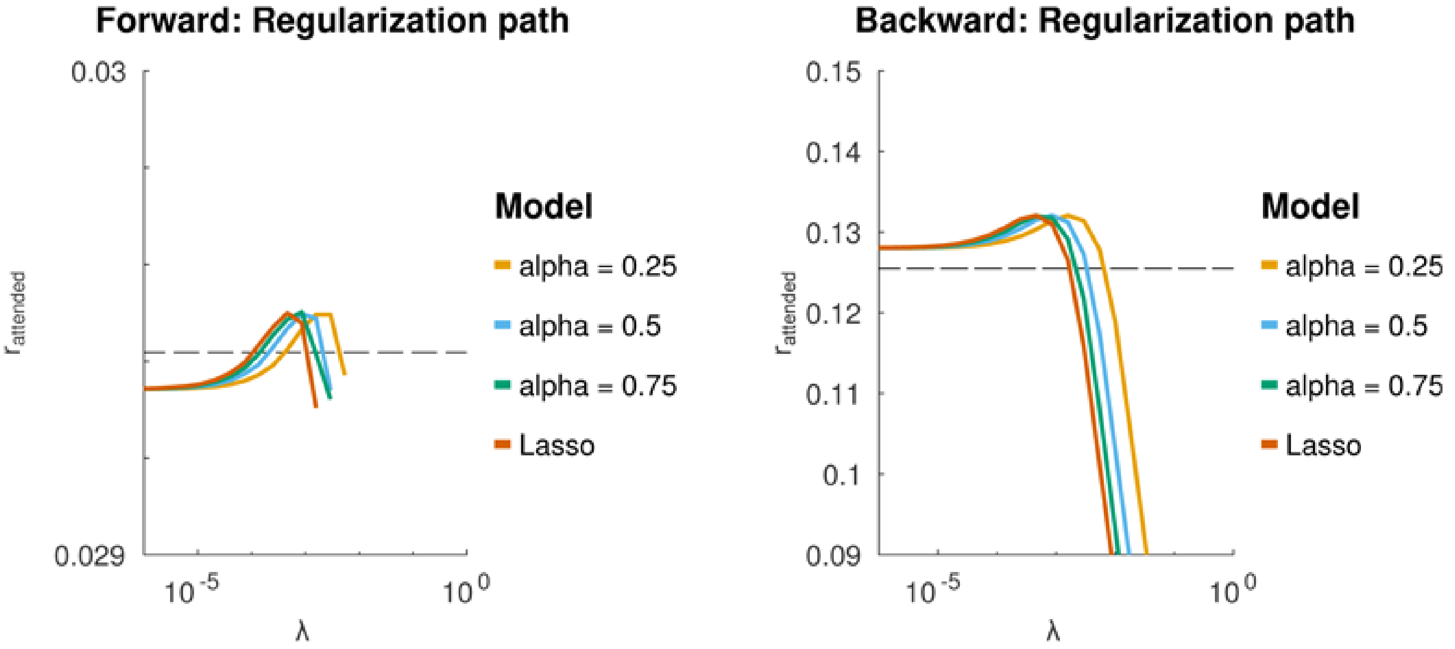

Figure A2. Group-mean validation-set regression accuracies obtained from forward/backward models with elastic net penalties. The elastic net has two tuning parameters, λ and α. The two panels show the group-mean validation set regression accuracies cross-validated over a relatively small grid of λ and α values. The prediction accuracies remain stable over a large range of λ values. The dashed line shows the regression accuracy for OLS.

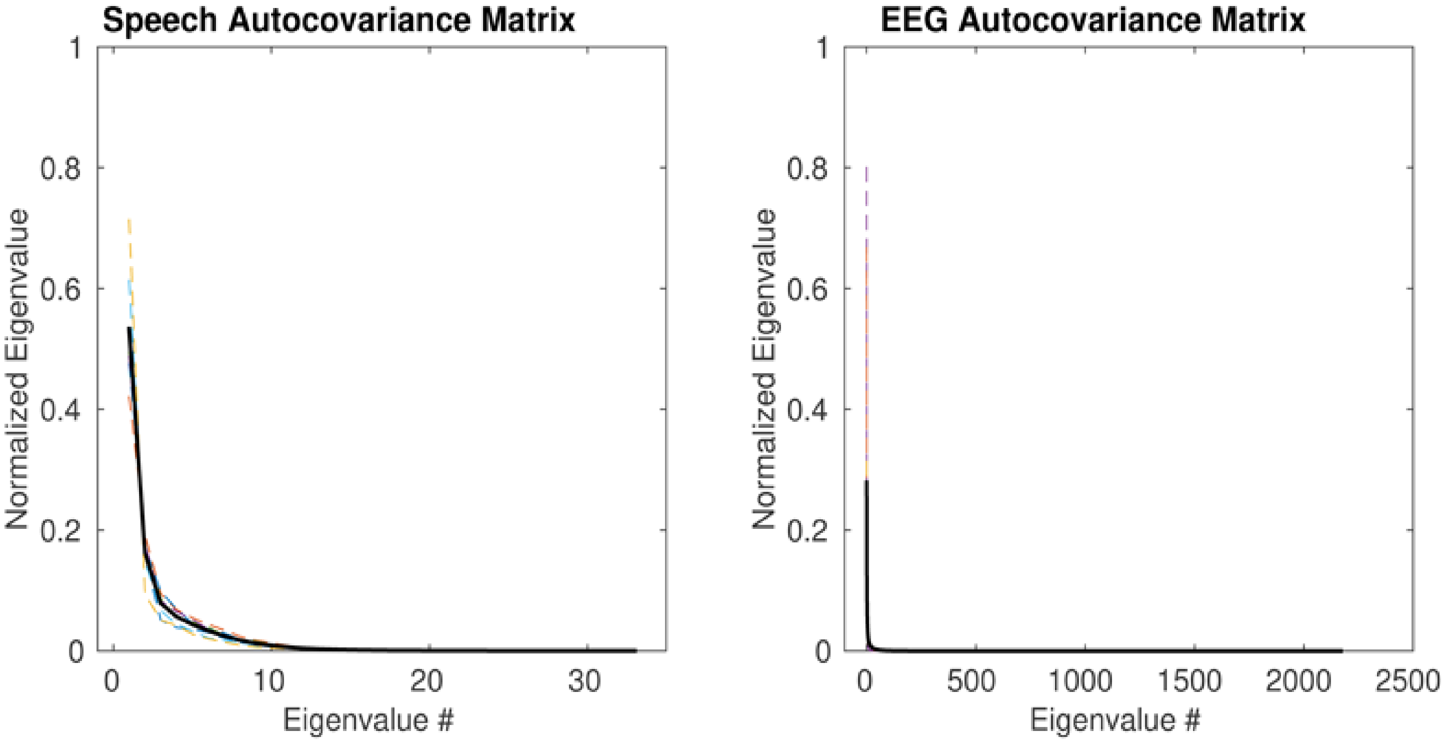

Figure A3. Normalized eigenspectra for speech and EEG autocovariance matrices ($\mathbf{X}^T\mathbf{X}$). Bold black line is the average across subjects. Individual subjects are shown as thin dashed lines.
